# Supplementary material for: Nucleotide-dependent switching and RIPb effector recognition of the barley susceptibility factor RACB
Source: Commun Biol. 2026 May 21;9:691. doi: 10.1038/s42003-026-10316-7 (PMC13195091; doi:10.1038/s42003-026-10316-7)
Supplement: Supplementary file 2 — Supplementary information [file 42003_2026_10316_MOESM2_ESM.pdf]

Supplementary Information to:

Nucleotide-dependent switching and RIPb effector recognition of the barley susceptibility factor RACB

Mariam Mohamadi[1], Mariem Bradai[2], Robert Janowski[3], Umut Günsel[1,3], Marie Tran[1], Sinika M. Kahl[1], Christopher McCollum[2], Dierk Niessing[3,4], Ralph Hückelhoven[2] and Franz Hagn[1,3,*]

[1] Bavarian NMR Center (BNMRZ) and Structural Membrane Biochemistry, Department of Bioscience, TUM School of Natural Sciences, Technical University of Munich, 85748 Garching, Germany

[2] Chair of Phytopathology, TUM School of Life Sciences, Technical University of Munich, 85354 Freising, Germany

[3] Molecular Targets and Therapeutics Center (MTTC), Institute of Structural Biology, Helmholtz Munich, 85764 Neuherberg, Germany

[4] Institute of Pharmaceutical Biotechnology, Ulm University, 89081 Ulm, Germany

*Corresponding author. E-mail: franz.hagn@tum.de, Tel: +49-89-289-52624

Supplementary Notes

Supplementary Note 1

RACBwt (Uniprot ID Q8RW50)

M_1SASRFIKCVTVGDGAVGKTCMLISYTSNTFPTDYVPTVFDNFSANVVVDGNTVNLGLWDT
AGQEDYNRLRPLSYRGADVFLLAFSLISKASYENVSKKWIPELKHYAPGVPIILVGTKLDLRD
DKQFFVDHPGAVPITTAQGEELKKLIGAPYYIECSSKTQLNVKGVFDAAIKVVLQPPKAKKKK
KAQRGACSIL$_{197}$

RIPb-CC2 (421-612, NCBI ID XP_044955000.1:

MQNSKTSRNGSSDAPQRTSPATPRSSRVAKTGGNETDSAGITPTRTPTERSPKVIERRSPR
SPVTEKKRPSRLTELDSKVNQLQDELKKTKEQLSASEARRCQAQQEAEEAKKQGQDVSLKL
EESQCQLVNLSAAEESRLQELRKIQQERDRTWQAELEAFQKQQSVDAAALSSALSEIQRLK
LQLEVTVQSDTARAKQCEHADSELEALKQEMELRLATIEALKVNISESDKAAADANAMATET
KLQLETAKATIDTLLAEGARLQECLKSKDIELGESKARVVALEEDLKKAHAAGNEILDEAQAG
NANGGFGSPLTEVLKKSPHPTSDINGSPDPEIEHLRMALEVAEMRYQEEQTRLTFETKTVYE
MLENVKSECTRQVCDIELKLKSKNDELMAAQAALTGKAQEDLHRSDGLSE$\mathbf{M}_{421}$QPELEAKL
MKSITDIAELKANLMDKENALQSLVEENETLKSEAGKKEADVQQRYEAAVAELELAKAAEQD
VRMRLGYVTEEADKSSRRAARASEQLDAAQAASTEADAELRRLRVQSDQWRKAAEAAAAA
LAGGGNNGGRMVERTGSLDTEYNGSIGGKLMGSPFSDEESPKRRNSGVLRRMSGLWKKG
P$\mathbf{K}_{612}$

Supplementary Tables

Supplementary Table 1: MD simulation setup parameters

Parameter	RACB-GDP	RACB-GMPPNP	RACB-GTP-RIPb-CC2 membrane complex
Simulation time (μs)	10	10	0.4
Number of independent runs	3	3	1
Timestep (fs)	2	2	2
Lipids per leaflet	n/a	n/a	632 POPC, 600 POPG
Temperature (K)	303.15	303.15	303.15
Pressure (bar)	1	1	1
Unit cell shape and size (Å)	Cubic: 75 x 75 x 75	Cubic: 78 x 78 x 78	Cubic: 203 x 203 x 157
Total number of atoms	39517	44327	608935
Number of water molecules	12209	15056	145903
Salt concentration (KCl)	150 mM	150 mM	150 mM
Simulation mode	NPT	NPT	NPT
Forcefield	CHARMM36m	CHARMM36m	CHARMM6m
Software	Gromacs 2025.2 CUDA	Gromacs 2025.2 CUDA	Gromacs 2025.2 CUDA

Equilibration for water box simulation

Force constants	Equilibration (125 ps, 1 fs timestep)
Protein backbone	1.0
Protein side chains	0.1
Water	0
Ions	0

Equilibration for membrane simulation

Force constants	Cycle 1 (125 ps, 1 fs timestep)	Cycle 2 (125 ps, 1 fs timestep)	Cycle 3 (125 ps, 1 fs timestep)	Cycle 4 (500 ps, 2 fs timestep)	Cycle 5 (500 ps, 2 fs timestep)	Cycle 6 (500 ps, 2 fs timestep)
Protein backbone	10.0	5.0	2.5	1.0	0.5	0.1
Protein side chains	5.0	2.5	1.0	0.5	0.1	0.0
Water	2.5	2.5	1.0	0.5	0.1	0.0
Lipid tails	2.5	2.5	1.0	0.5	0.1	0.0
Lipid head groups	2.5	2.5	1.0	0.5	0.1	0.0
Ions	10.0	0.0	0.0	0.0	0.0	0.0

Supplementary Figures

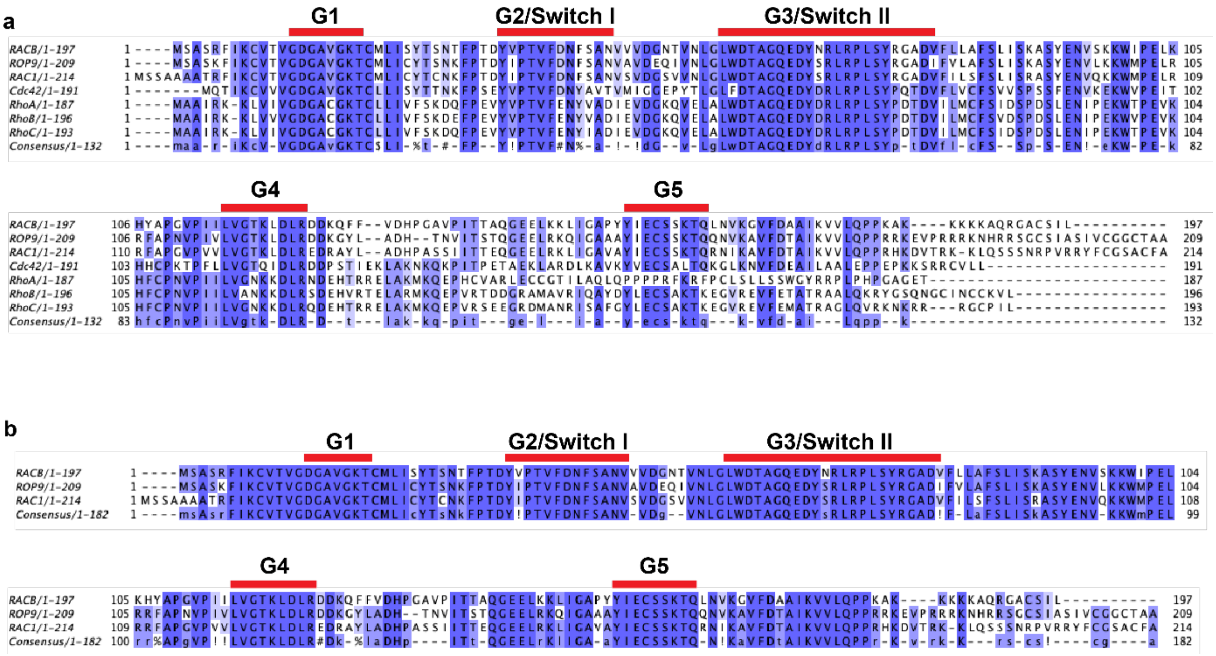

Supplementary Figure 1. Multiple Sequence alignment of RACB and other plant and human small GTPases. (a) Proteins from top to bottom: Barley RACB, *Arabidopsis thaliana* ROP9, *Oryza sativa* RAC1, human Cdc42, human RhoA, human RhoB, human RhoC. (b) same as in (a) but with only the plant proteins RACB, ROP9 and RAC1.

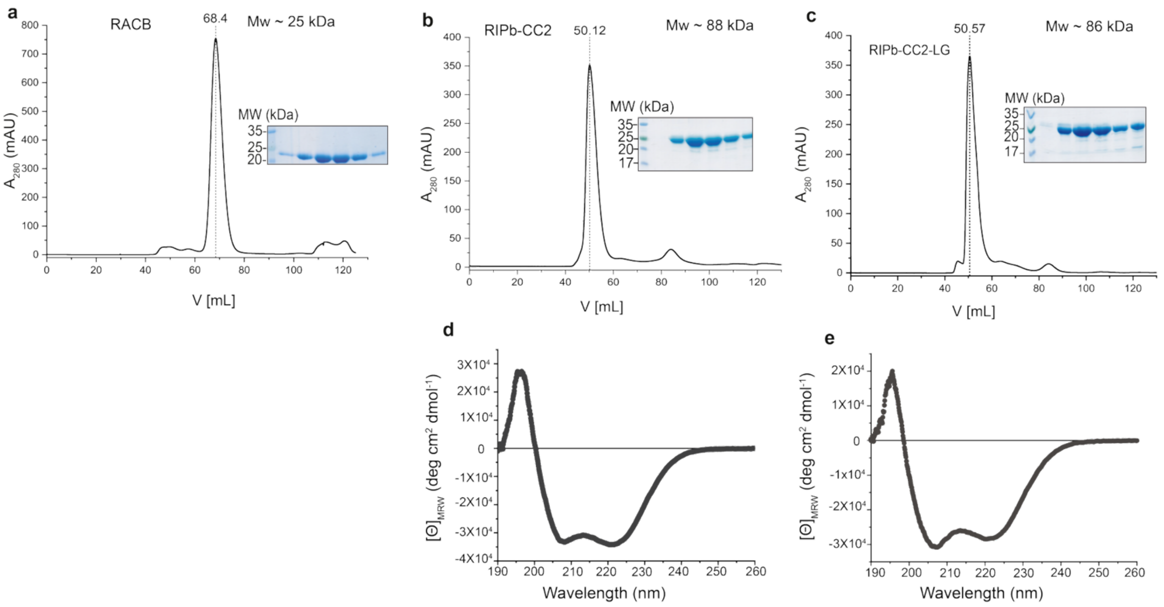

Supplementary Figure 2. Purification and biophysical characterization of RACB and RIPb-CC2 variants. (a-c) Size-exclusion chromatography (SEC) profiles using a Superdex™ 75 pg HiLoad 16/600 column (Cytiva) and corresponding SDS-PAGE analyses of the final purification step for (a) RACB, (b) RIPb-CC2 and (c) the RIPb-CC2 Q540L/W541G double variant. The estimated peak protein concentration on the SEC column was (a) 35 µM (b) 23 µM (c) 36 µM. All proteins were purified to homogeneity, and peak fractions containing the target proteins were pooled and concentrated. The lower elution volumes for the two RIPb-CC2 constructs with an apparent MW of 88 or 86 kDa suggests a dimeric assembly. Far-UV circular dichroism (CD) spectra of (d) RIPb-CC2 and (e) RIPb-CC2 Q540L/W541G double variant, exhibiting the characteristic minima at 208 and 222 nm, indicative of an α-helical secondary structure.

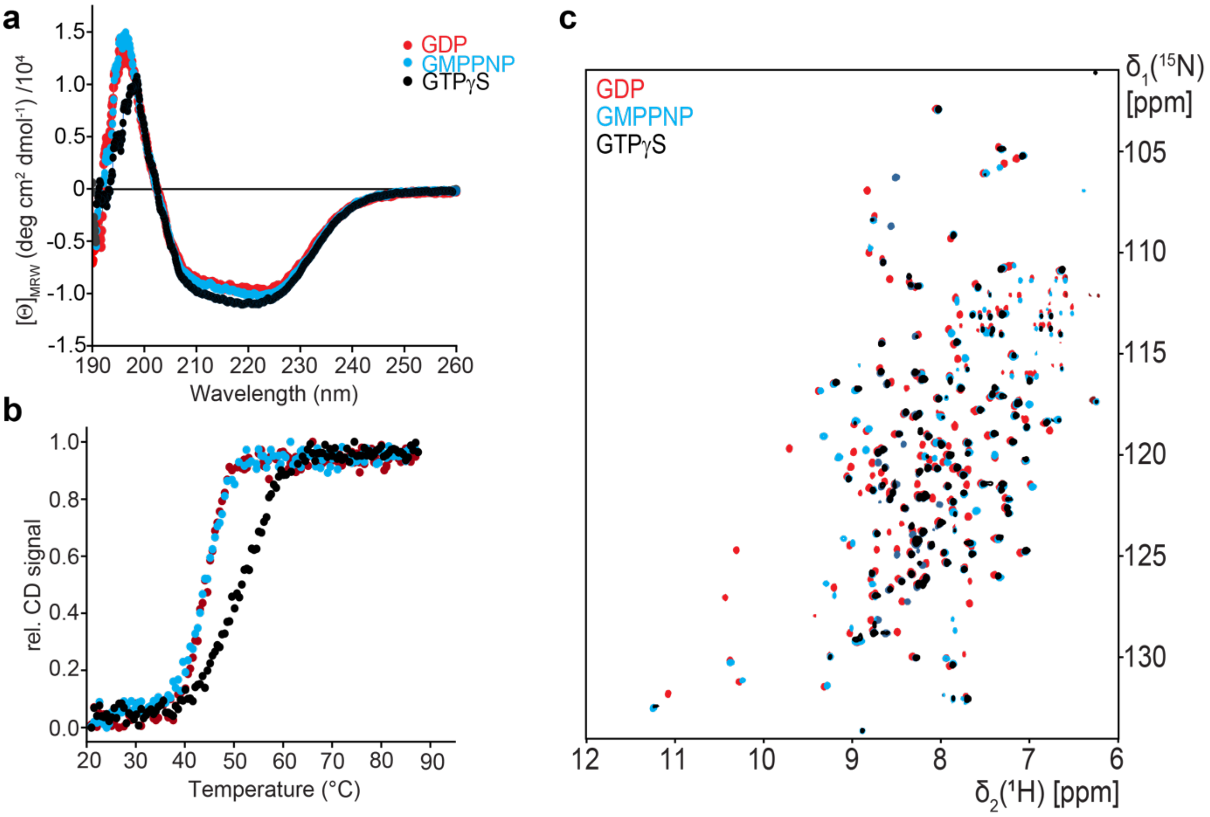

Supplementary Figure 3. Biophysical characterization of barley RACB in its nucleotide-bound states. (a) Circular dichroism (CD) spectra of RACB in the presence of GDP (red), GMPPNP (blue), or GTPγS (black) show a mixture of α-helical and ß-sheet secondary structure in all nucleotide-bound states. (b) Thermal melting profiles of RACB monitored by CD at 222 nm. The detected melting temperature (T_m) is 44 °C for the GDP and GMPPNP-bound states, and 52 °C in complex with GTPγS. (c) Overlay of 2D-[^{15}N,^{1}H]-HSQC NMR spectra of RACB bound to GDP (red), GMPPNP (blue), or GTPγS (black) at 25 °C.

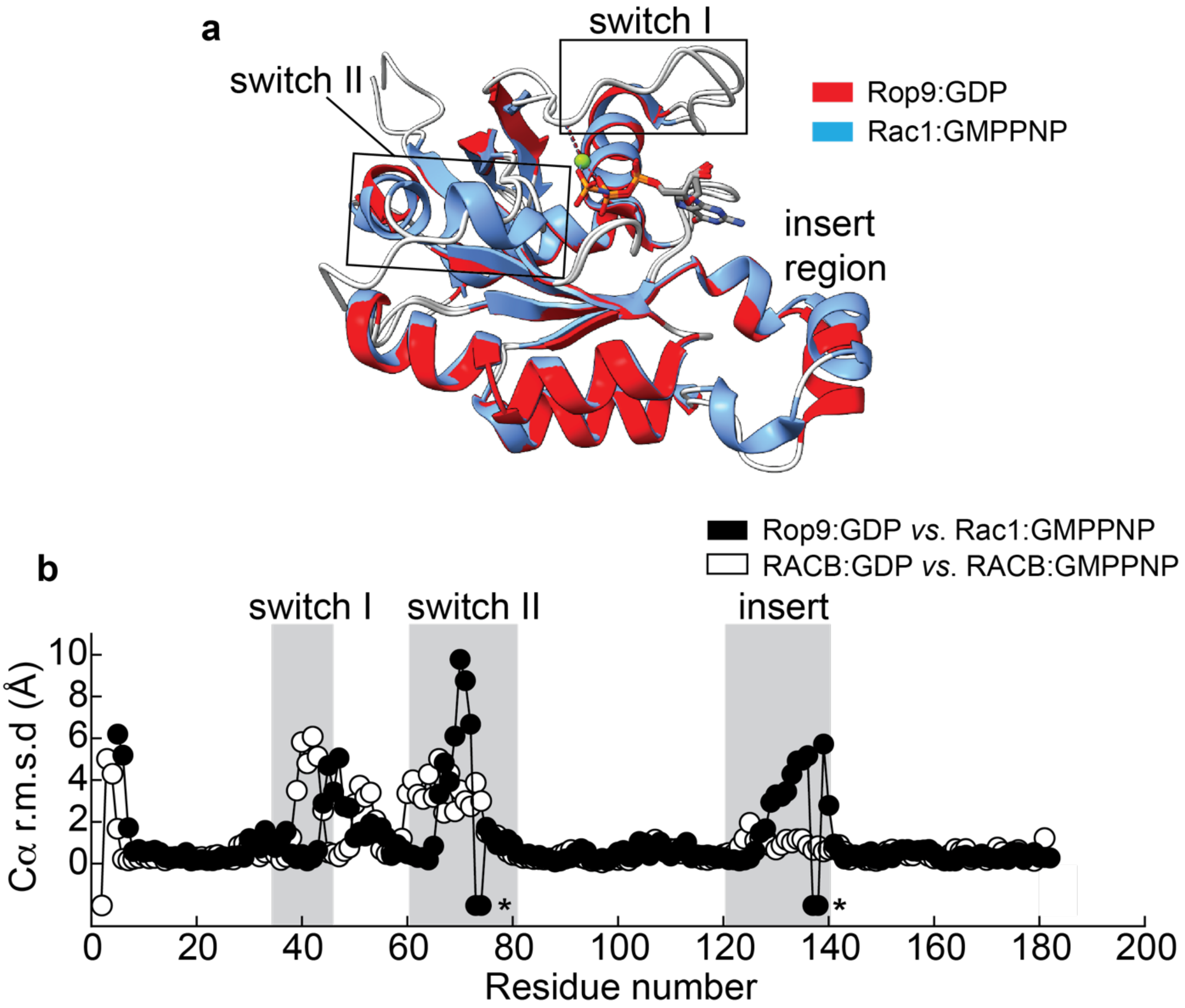

Supplementary Figure 4. Structural comparison of plant small GTPases in different nucleotide-bound states. (a) Superposition of *Arabidopsis thaliana* ROP9-GDP (red) and *Oryza sativa* RAC1-GMPPNP (blue) highlights conserved nucleotide-dependent rearrangements of the switch regions. (b) Cα displacement plot illustrating backbone deviations between GDP- and GMPPNP-bound RACB (white circles) and between Rop9-GDP and RAC1-GMPPNP (black circles). The most significant changes occur in switch I, II, and insert regions (gray boxes). Asterisks denote missing residues in one of the compared structures.

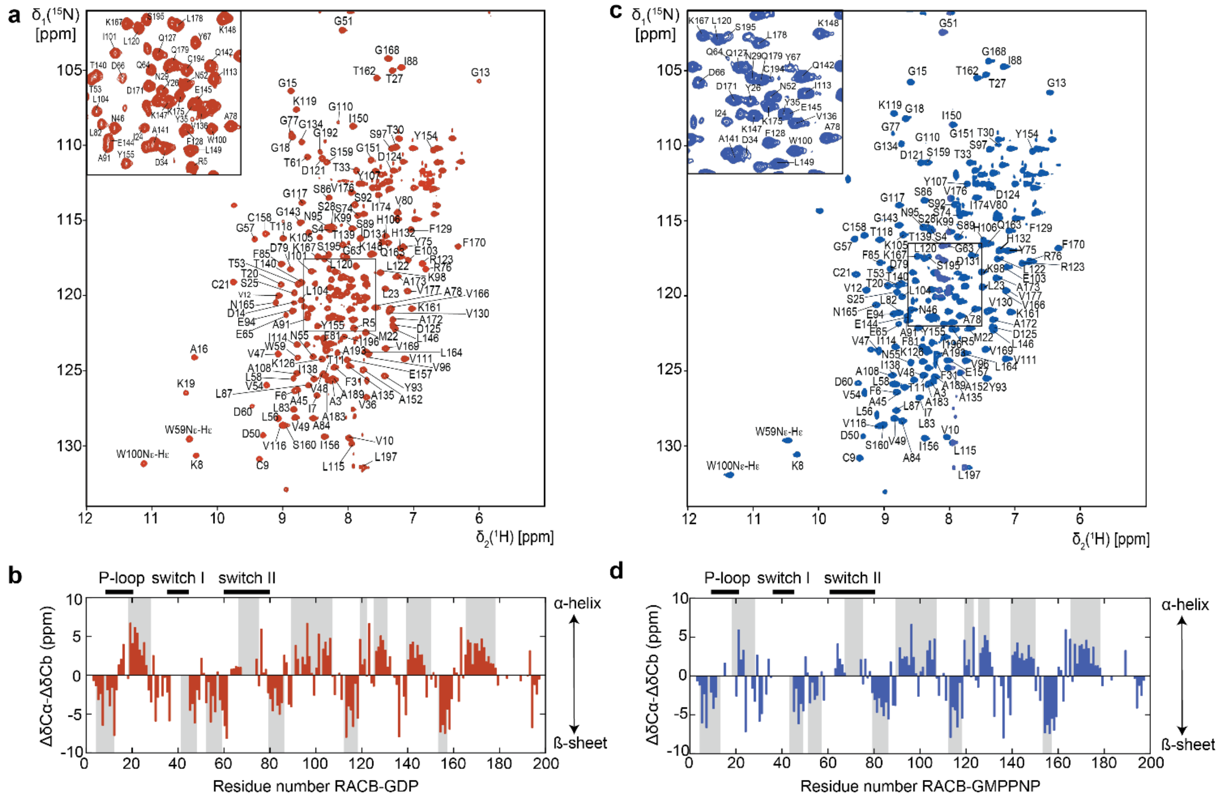

Supplementary Figure 5. NMR backbone resonance assignments of RACB in different nucleotide-bound states. (a) 2D-[^{15}N,^{1}H]-HSQC spectrum of RACB-GDP. (b) Secondary chemical shift (ΔδCα–ΔδCβ) for RACB-GDP. (c) 2D-[^{15}N,^{1}H]-HSQC spectrum of RACB-GMPPNP. (d) Secondary chemical shift (ΔδCα–ΔδCβ) for RACB-GMPPNP. Positive values in (b) and (d) indicate α-helical, negative values β-strand backbone geometry.

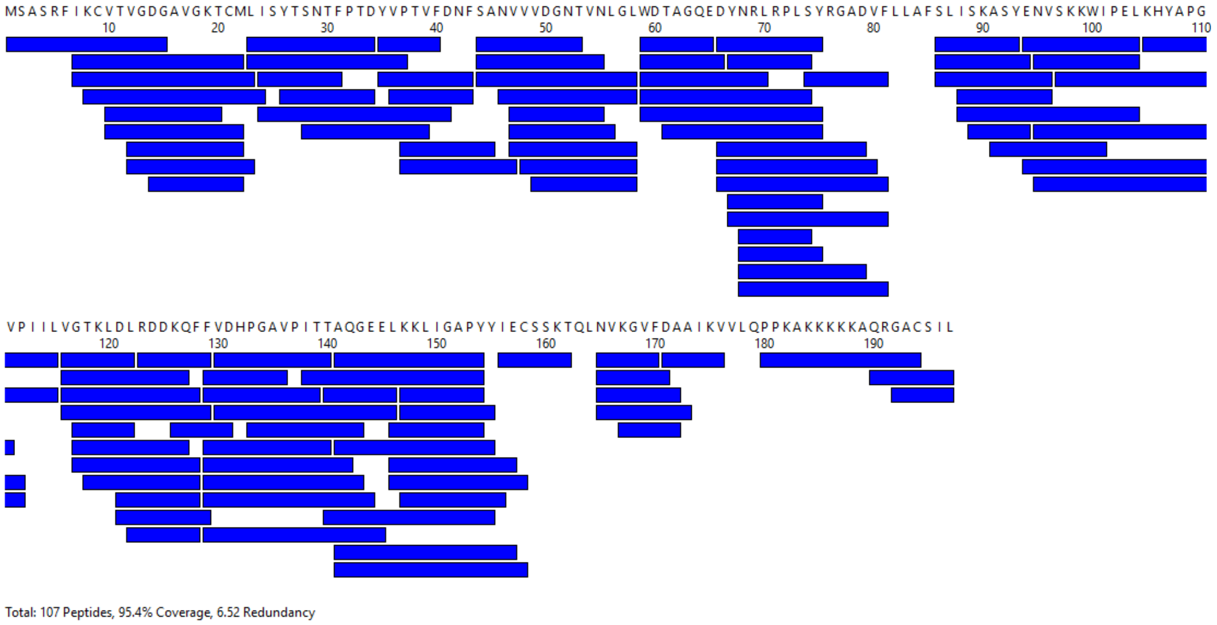

Supplementary Figure 6. Peptide coverage for HDX experiments of RACB in the GDP and GTPγS bound states. 107 peptides were detected resulting in 95.4% sequence coverage and a 6.52-fold redundancy.

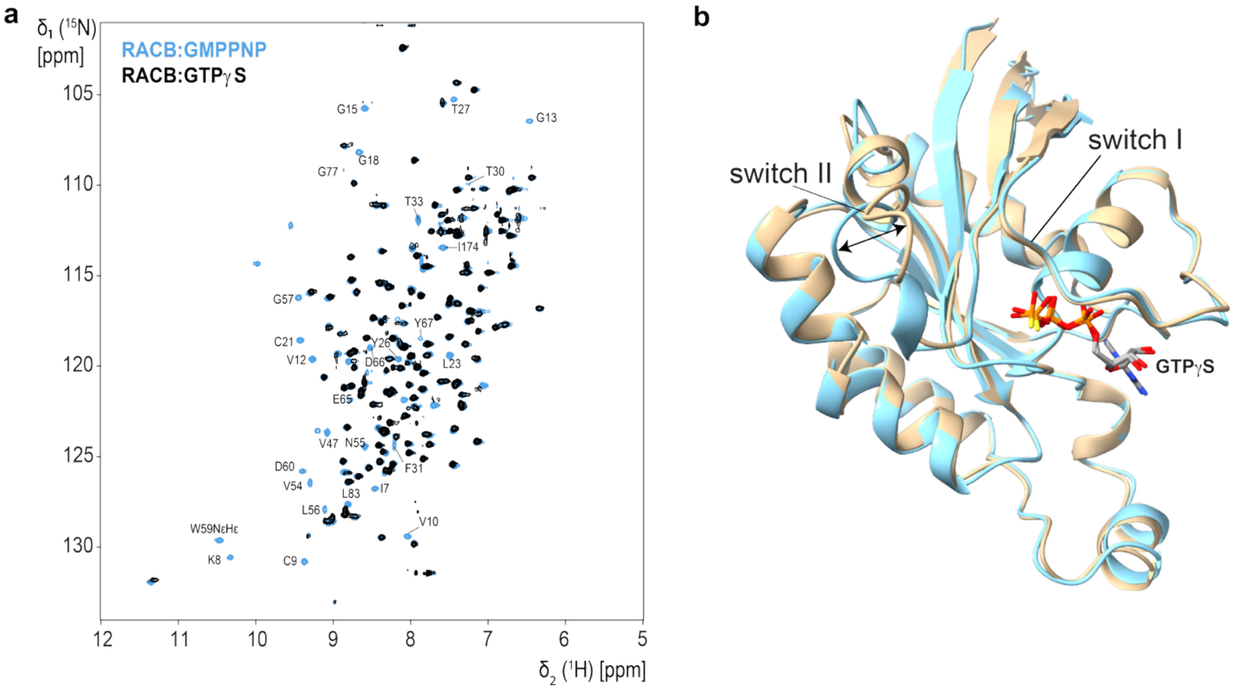

Supplementary Figure 7. Structural dynamics and line broadening of RACB in complex with GMPPNP and GTPγS probed by NMR and crystallography. (a) 2D-[^{15}N-^{1}H]-HSQC NMR spectra of uniformly ^{15}N-labeled RACB (0.1 mM) recorded at 30 °C in its GMPPNP-bound (blue) and GTPγS-bound (black) states. Several resonances undergo severe line broadening and disappear in the RACB-GTPγS spectrum. The assignments in the GMPPNP form of resonances missing in the GTPγS bound state are labeled. (b) Structural heterogeneity as seen in different monomers (e.g. between chains A and C) in the asymmetric unit of the crystal structure of RACB-GTPγS (pdb: 28NN) suggest structural heterogeneity, which might give rise to the NMR line broadening effects seen in solution (a).

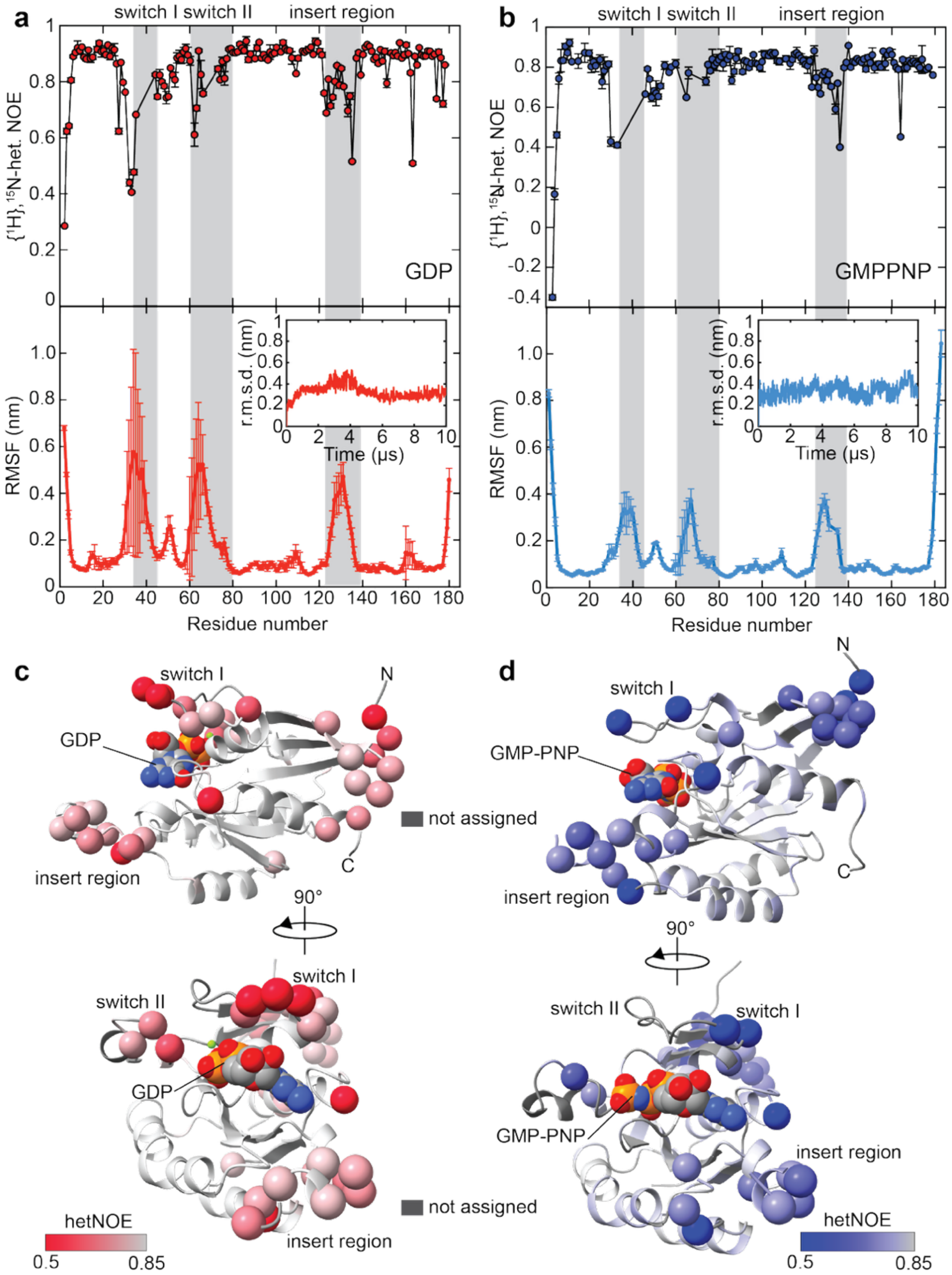

Supplementary Figure 8. NMR and MD analysis of fast dynamics in RACB in different nucleotide-bound states. (a) upper panel: {^{1}H}-^{15}N-heteronuclear NOE values of RACB in complex with GDP measured at 30 °C and 950 MHz ^{1}H frequency. Data are represented as mean value ± S.D. derived from n=2 technical replicates. Error bars are derived from two Lower panel: root mean squared fluctuations (RMSF) values for Cα atoms in RACB-GDP. The mean Cα atom fluctuations and the S.D. are shown derived from n=3 individual simulations with t=10 µs MD simulations at T=30°C. insert: root mean square deviation (r.m.s.d) of Cα coordinates during the simulation. (b) same as in (a) but with RACB-GMPPNP. (c) het NOE values of RACB-GDP color-coded onto the X-ray structure as indicated by the legend. (d) same as in (c) but with RACB-GMPPNP.

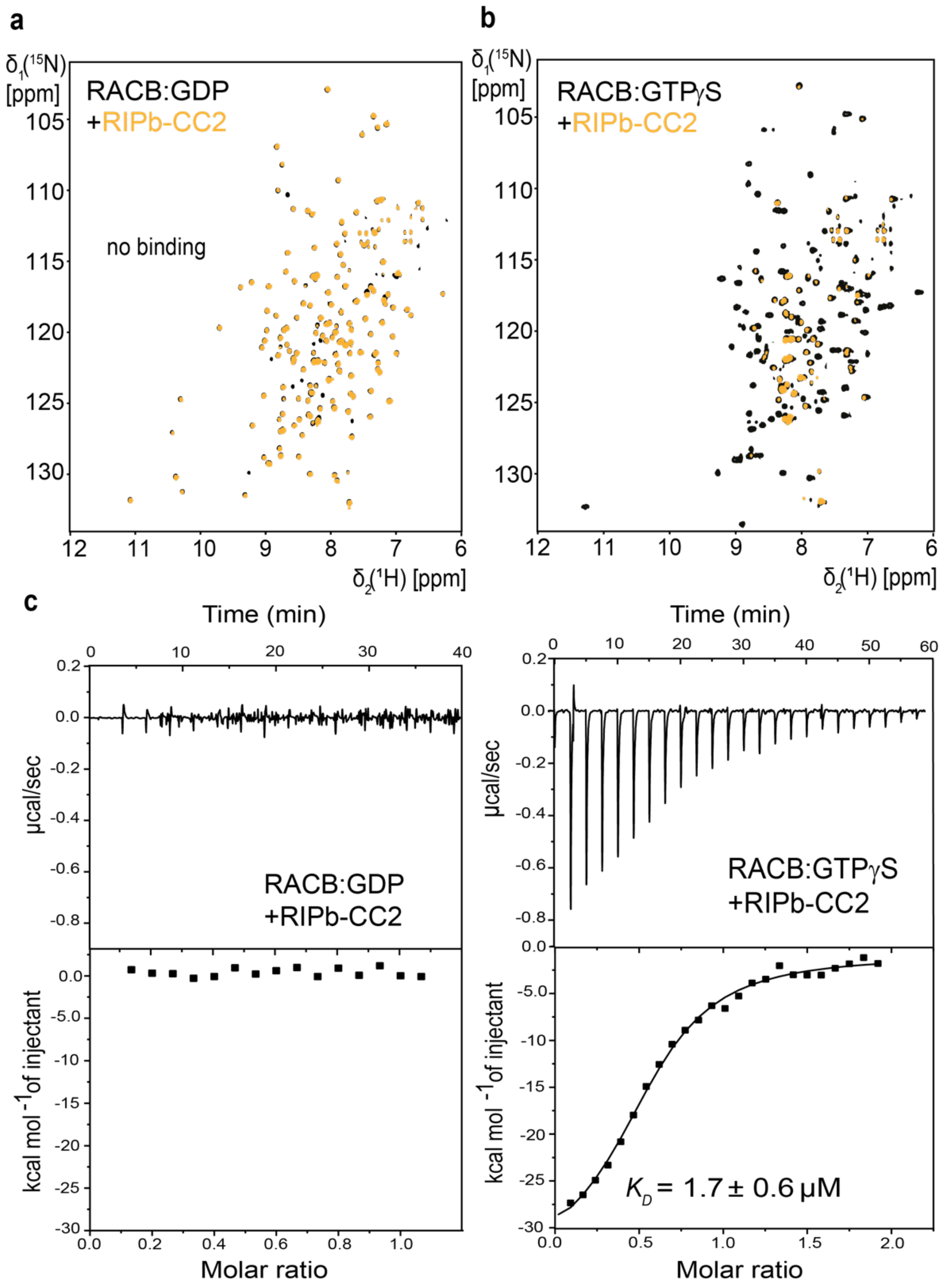

Supplementary Figure 9. Nucleotide-dependent interaction between RACB and RIPb-CC2. 2D-[^{15}N,^{1}H]-TROSY spectra of RACB-GDP (a) and RACB-GTPγS (b) spectra (black) alone and after the addition of a 1.2 molar excess of RIPb-CC2 (yellow). (c) Isothermal titration calorimetry (ITC) with RIPb-CC2 and RACB-GDP (left) or RACB-GTPγS (right) at T = 20°C. No binding was observed for the GDP-bound form, while RACB-GTPγS exhibited strong binding with the following parameters: K_D = 1.7 ± 0.6 μM, N (sites) = 0.7 ± 0.2, ΔG = -7.8 ± 0.2 kcal/mol, ΔH = -23 ± 8 kcal/mol, -TΔS = 16 ± 8 kcal/mol. Values denote the mean value and S.D. obtained from n=3 technical replicates.

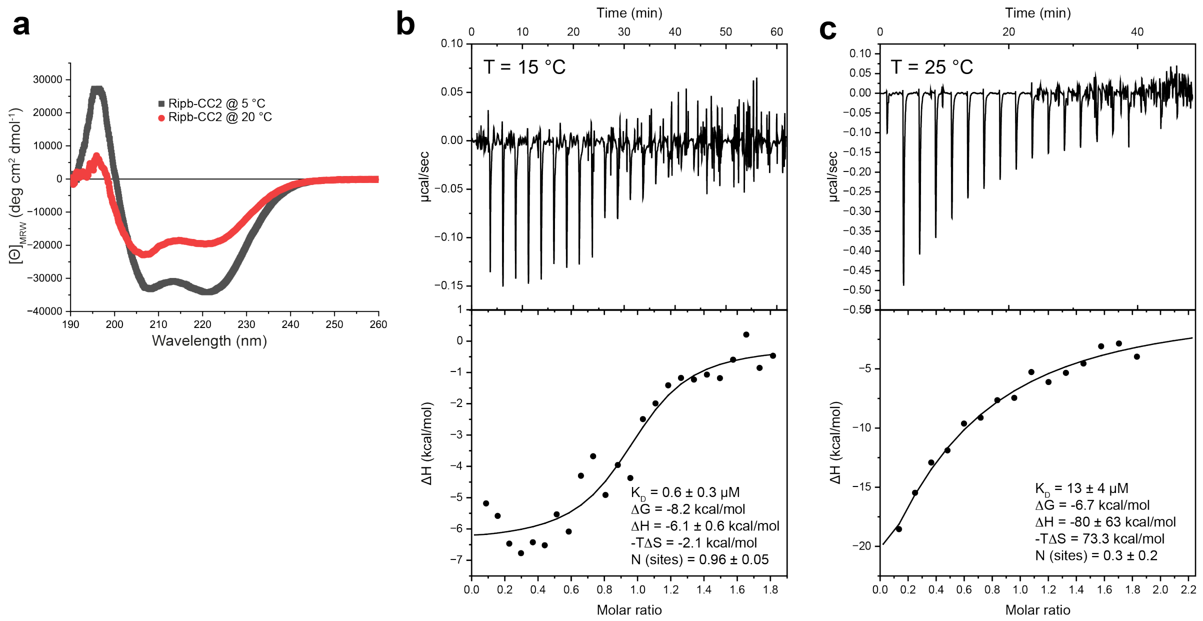

Supplementary Figure 10. Temperature-dependent folding state of RIPb-CC2 governs the affinity for RACB. (a) Far-UV CD spectra of RIPb-CC2 at 5 and 20°C indicating a marked increase in α-helical secondary structure content at the lower temperature. (b) ITC experiments with RACB-GTPγS and RIPb-CC2 at 15°C and (c) 25°C. The obtained binding data indicate higher affinity at lower temperature (0.6 *versus* 13 µM), as well as a 1:1 stoichiometry (n=0.96) at 15°C but only 0.3 (RIPb-CC2:RACB) at 25°C, and a very high binding enthalpy at 25°C of -80 kcal/mol *versus* only -6.1 kcal/mol at 15°C. Together these data indicate, consistent with the CD results in panel (a), that RIPb-CC2 does not adopt a fully folded α-helical state at 25°C but folds upon interaction with RACB.

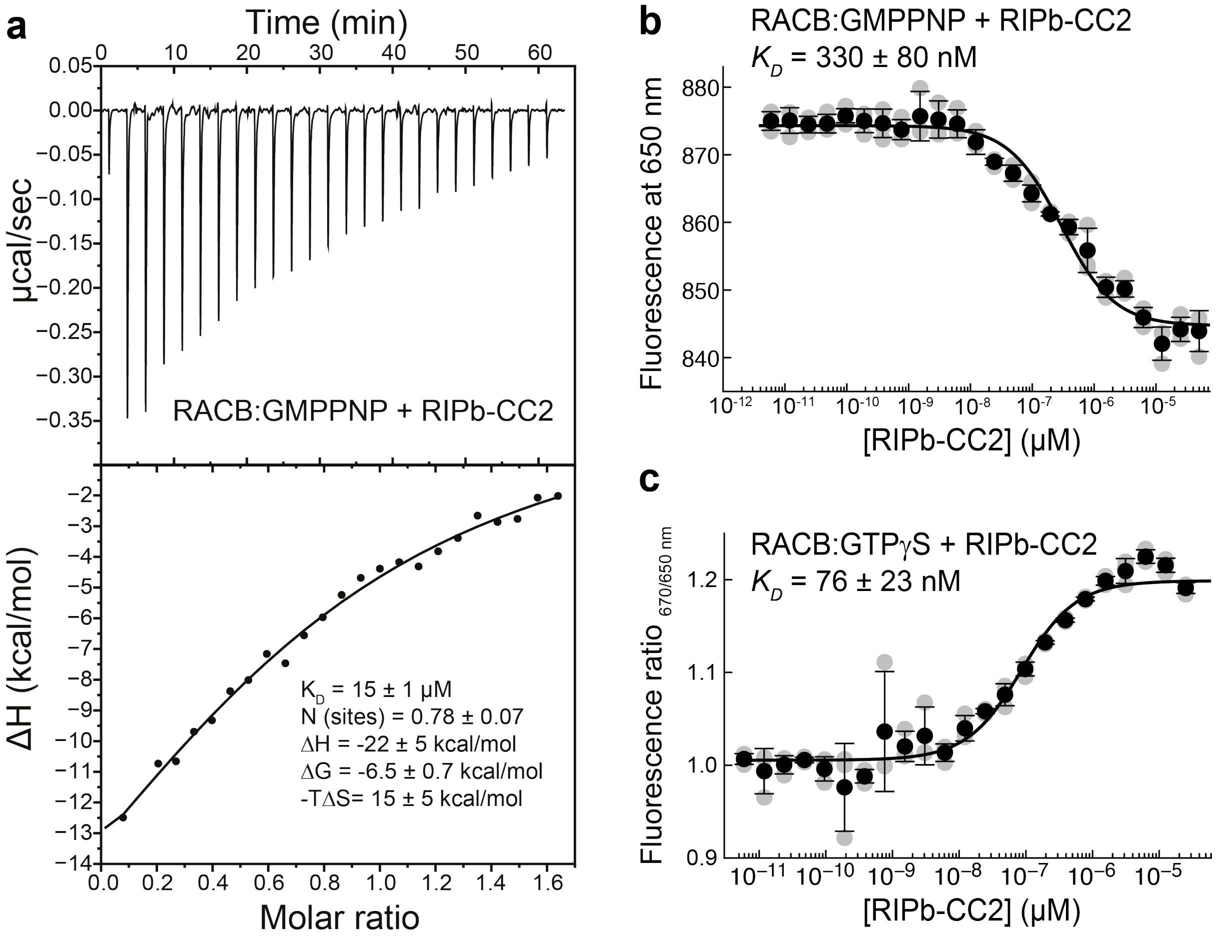

Supplementary Figure 11. ITC and microscale thermophoresis (MST) and spectral shift analysis of the RACB-RIPb-CC2 complex. (a) ITC with RACB-GMPPNP binding to RIPb-CC2 at T = 20°C showing a K_D value of 15 ±1 µM derived from n=3 technical replicates. (b) MST binding curve for RACB-GMPPNP titrated with RIPb-CC2, yielding a dissociation constant K_D of 330 ± 80 nM (c) Spectral shift data for RACB-GTPγS showing markedly tighter binding to RIPb-CC2, with a K_D of 76 ± 23 nM. Binding affinities were determined using NanoTemper analysis software and represent mean values and S.D. from n=3 technical replicates (individual data points shown in grey, average value and S.D. shown in black). After careful optimization of the assay, best quality data were obtained with MST for RACB-GMPPNP (panel b) and spectral shift (fluorescence intensity ratio) for RACB-GTPγS (panel c)[1].

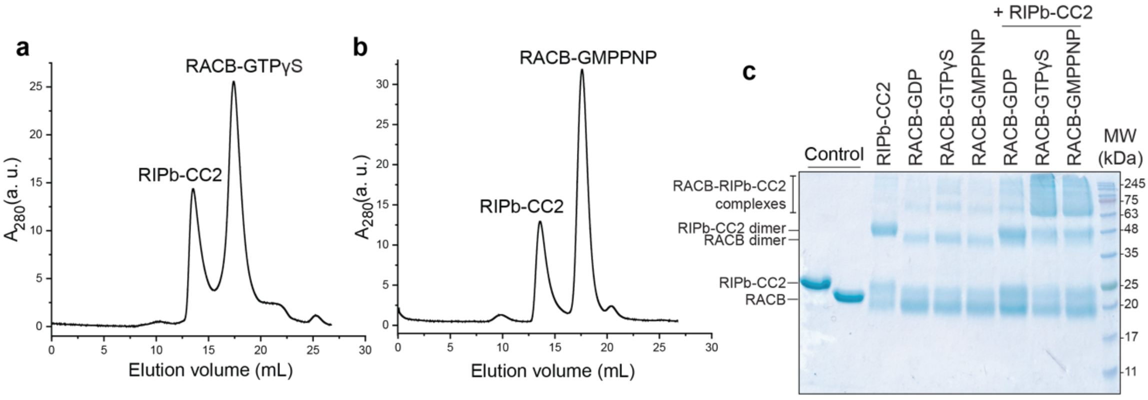

Supplementary Figure 12. Size exclusion chromatography (SEC) and chemical crosslinking of RACB with RIPb-CC2 in the GDP-, GMPPNP- and GTPγS-bound states. (a) A 1:1 mixture of RACB-GTPγS and RIPb-CC2 (20 µM each) preincubated for 15 min on ice was analyzed by SEC on a Superdex 200 10/300 GL column at T=4°C in 20 mM HEPES pH 7.5, 100 mM NaCl, 1 mM $MgCl_2$. The proteins dissociated during the SEC run, suggesting a binding affinity in the µM range. (b) Same as in (a) but with RACB-GMPPNP. The calculated MWs derived from column calibration for the two proteins are: RACB: 19.7 kDa, RIPb-CC2 (elongated dimer): 98.3 kDa. (c) SDS-PAGE analysis of BS^3-mediated crosslinking reactions between RACB and RIPb-CC2 using a 1:40 protein-to-crosslinker molar ratio. Reactions were performed with RACB bound to either GDP, GMPPNP or GTPγS as indicated. No crosslink between RACB and RIPb-CC2 was detected in the GDP-bound state, indicating the absence of complex formation. In contrast, clear higher-molecular-weight crosslinked bands appear with GMPPNP- or GTPγS-bound RACB, consistent with stable complex formation between active RACB and RIPb-CC2. A representative gel from n=3 independent technical replicates is shown. The shown gel figure is fully uncropped and unedited.

RACB:GTPγS + RIPb-CC2 (RIPb-CC2 sequence)

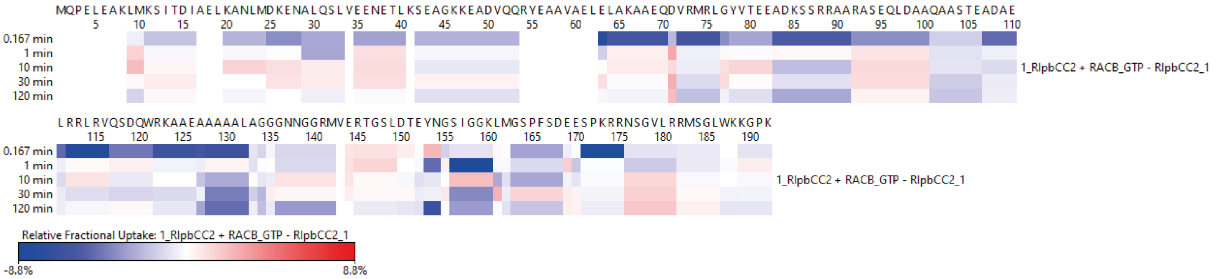

RACB:GTPγS + RIPb-CC2 (RACB sequence)

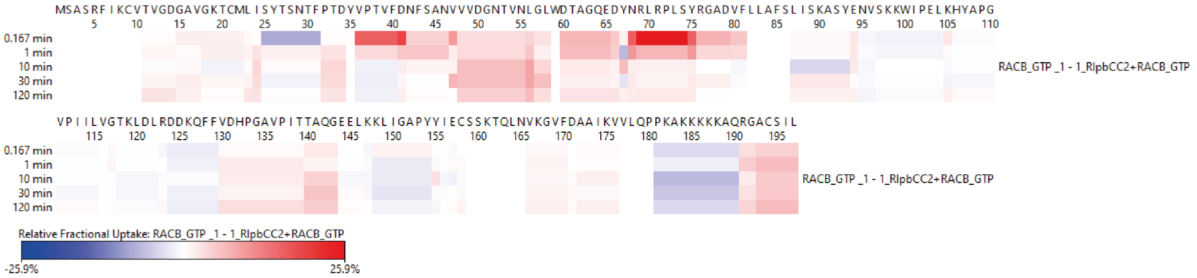

Supplementary Figure 13. HDX-MS deuterium uptake maps of the RACB–RIPb-CC2 complex. RACB–GTPγS and RIPb-CC2 were mixed in a 1:1 molar ratio before starting the HDX-MS process. Heat maps display the relative deuterium uptake, with the upper panel showing RIPb-CC2 peptide sequences and the lower panel showing uptake profiles for the full RACB sequence. For each protein, peptides are shown along the sequence from N- to C-terminus, and deuterium uptake is measured at different time points (10 s, 1 min, 10 min, 30 min, 120 min). Blue indicates reduced deuterium uptake (higher protection), while red indicates increased uptake (higher solvent exposure). Relative fractional uptake values are shown on the scale bars below each plot.

a

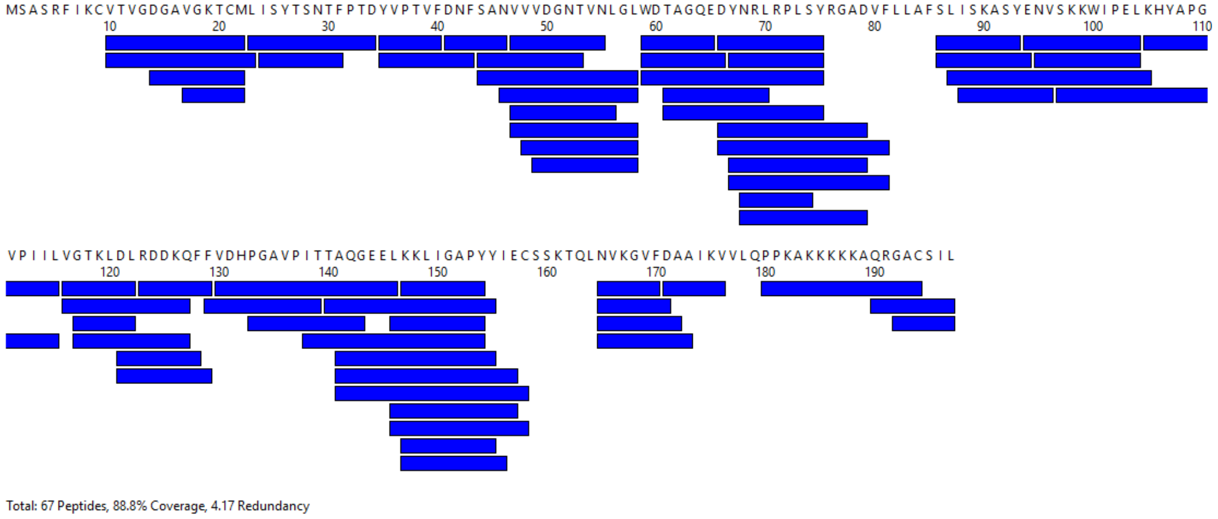

b

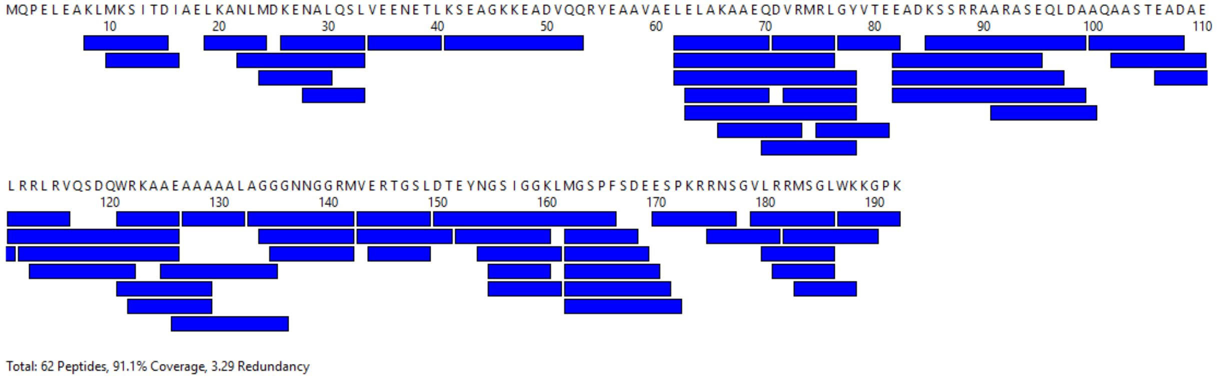

Supplementary Figure 14. Peptide coverage in HDX experiments of the RACB-GTPγS and RIPb-CC2 complex. (a) RACB sequence coverage: 67 peptides were detected resulting in 88.8% sequence coverage and a 4.18-fold redundancy. (b) RIPb-CC2 sequence coverage: 62 peptides were detected resulting in 91.1% sequence coverage and a 3.29-fold redundancy

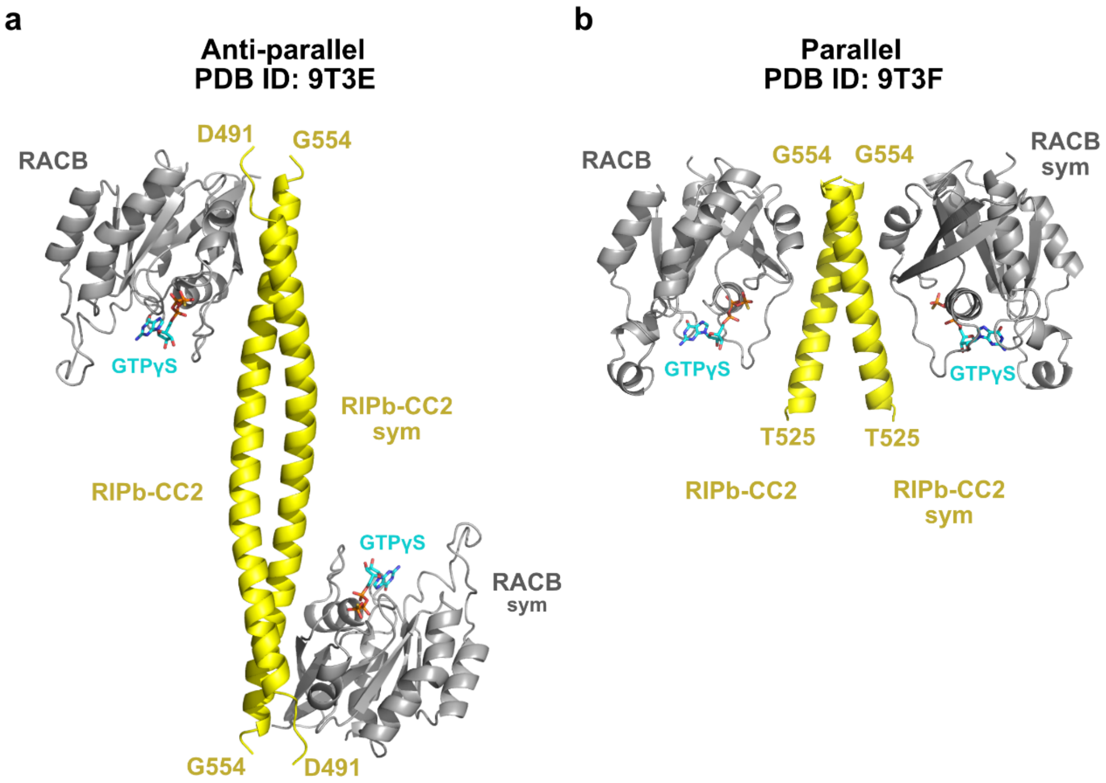

Supplementary Figure 15. Symmetry relations in the different X-ray structures of the RACB-RIPb complex. (a) Anti-parallel arrangement of the RIPb-CC2-C5 helices (yellow) in the 2.30 Å resolution RACB-GTPγS-RIPb-CC2-C5 complex structure (PDB ID: 9T3E). (b) Parallel orientation of the two copies of RIPb-CC2 in the 2.07 Å complex structure (PDB ID: 9T3F). In both panels the symmetry related molecules are labeled as 'sym'. The binding mode is identical in both structures.

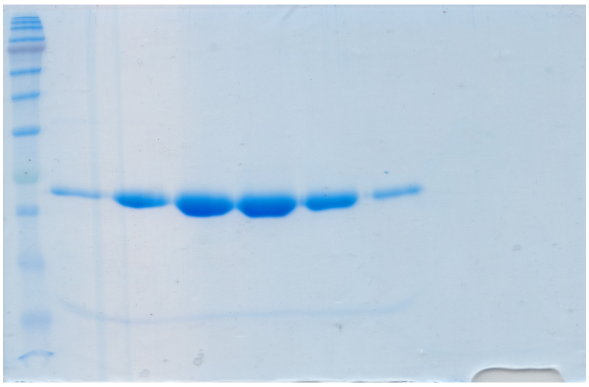

Supplementary Figure 16. Uncropped SDS-PAGE gel shown in Supplementary Figure 2a with SEC peak fractions of RACB. Molecular weight marker bands (in kDa) are (from top to bottom): 245, 180, 100, 75 (magenta), 63, 48, 35, 25 (green), 20, 17, 11.

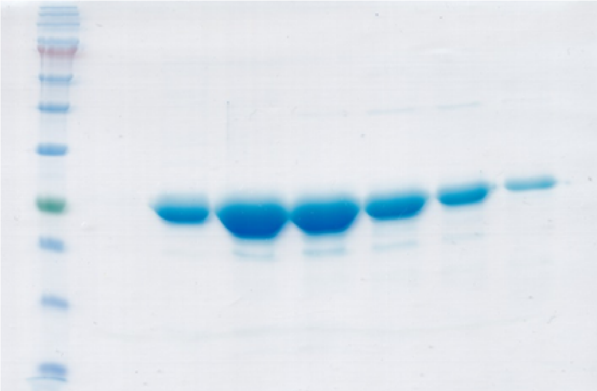

Supplementary Figure 17. Uncropped SDS-PAGE gel shown in Supplementary Figure 2b with SEC peak fractions of RIPb-CC2 wt. Molecular weight marker bands (in kDa) are (from top to bottom): 245, 180, 100, 75 (magenta), 63, 48, 35, 25 (green), 20, 17, 11.

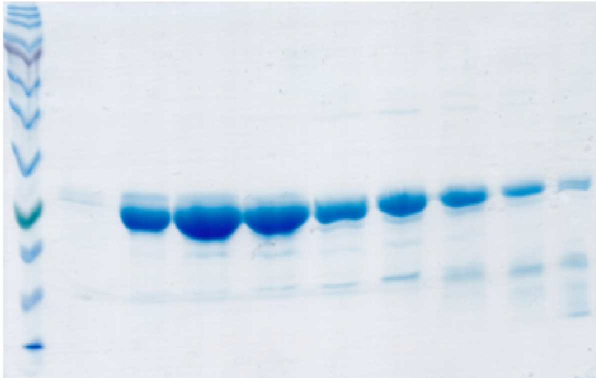

Supplementary Figure 18. Uncropped SDS-PAGE gel shown in Supplementary Figure 2b with SEC peak fractions of RIPb-CC2 Q540L/W541G. Molecular weight marker bands (in kDa) are (from top to bottom): 245, 180, 100, 75 (magenta), 63, 48, 35, 25 (green), 20, 17, 11.

Supplementary References

1 Nowak, J. S. *et al.* Microscale thermophoresis (MST) and spectral shift (SpS) in drug discovery. *TrAC Trends in Analytical Chemistry* **176** (2024). https://doi.org:10.1016/j.trac.2024.117716
