# Supplementary material for: Nucleotide-dependent switching and RIPb effector recognition of the barley susceptibility factor RACB
Source: Commun Biol. 2026 May 21;9:691. doi: 10.1038/s42003-026-10316-7 (PMC13195091; doi:10.1038/s42003-026-10316-7)
Supplement: Supplementary file 3 — Description of Additional Supplementary files [file 42003_2026_10316_MOESM3_ESM.pdf]

Description of Additional Supplementary files

File name: Supplementary Data

Description: All other raw data used for figure preparation are available as Supplementary Data.
